# Carrier—Different Significances of the Term

**Published:** 1915-03

**Authors:** 


**A Monthly Review of Medicine and Surgery**

EDITOR AND PUBLISHER, DR. A. L. BENEDICT, 228 Summer St., corner of Elmwood Ave., Buffalo, (Address for all communications. Please make personal and telephone calls before 1 P. M.)

Third, in age, on the western continent. Independent, but supports professional organization. News limited mainly to Western N. Y. and Penn.

Subscription $2.00 a year; $3.00 for two years in advance. Changes and errors in address or failure or duplication of delivery, should be reported immediately.

Yearly Volume 70. MARCH, 1915. No. 8

### Carrier—Different Significances of the Term.

We note that among 2400 prisoners at Bilibid Prison, Manila, 84 were cholera carriers. Of these 4 developed the disease in 4-18 days after their discovery. When we reflect upon the use of the term as regards diphtheria, typhoid and other diseases, it is apparent that it is used in at least three different senses:

1. The germ is present, the disease being in a state of incubation, as in the four cases mentioned;

2. The germ is present, usually during an epidemic or when a disease is widely endemic but the carrier does not develop the disease, either on account of immunity of some form or on account of general resistance, accidental failure of the germs to secure efficient inoculation or for some other reason;

3. The germs persist as harmless parasites after recovery from the disease, the immunity being more or less temporary or else practically permanent as in typhoid or other semelincident disease;

4. To these varieties, may possibly be added a type in which more or less permanent colonization of germs occurs, in a vegetative way, without previous occurrence of the disease and without definite immunity or resistance.

We use the non-committal word germ to include both bacterial and animal organisms of widely different kinds. It seems to us that expert study might be devoted to the consideration of essential and non-essential differences among these types, to the establishment of general principles and to the adoption of an exact classification and nomenclature.

